# Investigation of a new graphene strain sensor based on surface plasmon resonance

**DOI:** 10.1038/s41598-020-73834-2

**Published:** 2020-10-09

**Authors:** Zenghong Ma, Zijian Chen, Jian Xu, Weiping Li, Lian Zhang, Lei Wang

**Affiliations:** 1 Basic Experimental and Training Center, Tianjin Sino-German University of Applied Sciences, Tianjin, 300350 People’s Republic of China; 2 New Energy Department, Tianjin Sino-German University of Applied Sciences, Tianjin, 300350 People’s Republic of China; 3 College of Physics and Electronic Engineering, Xinyang Normal University, Xinyang, 464000 People’s Republic of China; 4 School of Physics and Optoelectronic Engineering, Xidian University, Xi’an, 710071 People’s Republic of China

**Keywords:** Nanophotonics and plasmonics, Optical sensors

## Abstract

The high confinement of surface plasmon polaritons in graphene nanostructures at infrared frequencies can enhance the light-matter interactions, which open up intriguing possibilities for the sensing. Strain sensors have attracted much attention due to their unique electromechanical properties. In this paper, a surface plasmon resonance based graphene strain sensor is presented. The considered sensing platform consists of arrays of graphene ribbons placed on a flexible substrate which enables efficient coupling of an electromagnetic field into localized surface plasmons. When the strain stretching is applied to the configuration, the localized surface plasmon resonance frequency sensitively shift. The strain is then detected by measuring the frequency shifts of the localized plasmon resonances. This provides a new optical method for graphene strain sensing. Our results show that the tensile direction is the key parameter for strain sensing. Besides, the sensitivity and the figure of merit were calculated to evaluate the performance of the proposed sensor. The calculated figure of merit can be up to two orders of magnitude, which could be potentially useful from a practical point of view.

## Introduction

Since its first isolation by Geim and Novoselov in 2004^1–3^, graphene has attracted a huge amount of research due to its unique electrical and mechanical properties, such as ultra-high mobility^4^, transparency^2^, Young modulus^5^, etc. It has been reported that as a new material, graphene has broad application prospects in the field of effective transistor^6^, transparency conductive film^7^, clean energy equipment^8^, sensors^9–13^, and so on. Especially, it has been reported that graphene is extremely sensitive to external strain stimuli^5,14^, so the ever-increasing interest in graphene is driven by its potential for developing strain sensors. Strain sensors can be used to measure local deformations and are mainly used for damage detection, structure characterization and fatigue studies of materials. Compared with other material based strain sensors, the ultra-thin transparent graphene devices are more commercial and easily integrated, which has attracted much attention for their potential applications in miniaturized and high capability strain sensors. In recent years, different types of graphene-based strain sensors have been developed, including resistance-type graphene strain sensors^9,15–19^, capacitive strain sensors^20–22^, fiber-optic graphene strain sensors or optical strain sensors deal with the plasmonic-enhanced Raman spectrum^23–26^, in-plane or tunneling graphene strain sensors^27^ and other graphene strain sensors^28–31^. Among the abovementioned strain sensors, the graphene-based piezoresistive sensors have become the most commonly used electromechanical sensors with relatively simple read-out systems. But the resistance-type graphene strain sensors are susceptible to electromagnetic interference from nearby instruments and charged objects. On the other hand, even if highly sensitive strain sensors can be obtained through the above methods, the strain applied is relatively large and not easy to achieve in practice. Besides, large stresses can also cause unrecoverable deformation of graphene, which greatly limits the applications of graphene strain sensors. Therefore, it is necessary to explore a new way to realize the high sensitive strain sensing of graphene without substantially destroying its crystal structures.

Due to the single atomic layer thickness of graphene, the optical response of graphene can be easily tuned by strain engineering^32^. Under strain engineering, the two carbon sublattices of graphene can be inequivalent and the electrical conductivity changed^33–36^, making graphene as a good candidate in optical-based strain applications^37^. Surface plasmon resonance (SPR) strain-sensors are typical optical sensors. In this paper, we explored a type of graphene strain sensors based on localized graphene plasmon polaritons, which has little relevant literature been reported so far. The considered sensing platform consists of a periodic array of graphene nanoribbons (PAGRs) placed on flexible substrate PDMS. When the strain engineering is applied to the platform, the in-plane conductivity of graphene will be anisotropic, which gives rise to the frequency shift of the plasmon resonance. Besides, we also studied in detail how the relative shift in plasmon frequency $\Delta f$ between strained and unstrained graphene varies with the graphene parameters, including the width *W* of the graphene ribbons, the angle $\theta$ between the zigzag and the tension direction, Fermi energy $E_F$ and strain modulus $\kappa$. In further, we show that the value of $\Delta f$ depends more on the angle $\theta$ and the strain modulus $\kappa$ than others. This phenomenon can be understood by the anisotropic optical conductivity and the localized plasmon resonance on graphene under strain engineering. Besides, the sensitivity and the figure of merit (FOM) were calculated to assess the performance of the proposed sensor. Compared with previously reported graphene strain sensors, the proposed localized plasmon resonance-based strain sensors exploit frequency shifts of the plasmonic resonances. The resonance position is very sensitive to changes in the conductivity of graphene when strain stretching is applied. On the other hand, our results indicate that our proposed sensing mechanism is sensitive only to the direction in which strain stretching is applied, but not to other parameters such as Fermi energy, size of the structure, and so on. Besides, Even for small deformation and low graphene quality, relatively high FOM factors can be obtained in the proposed strain sensor. This will be very beneficial for the realization of strain sensing. Moreover, the proposed configuration in the manuscript is quite simple in structure and easy to realize in an experiment, which provides a different route toward ultra-sensitive strain sensors.

## Results

**Figure 1:**
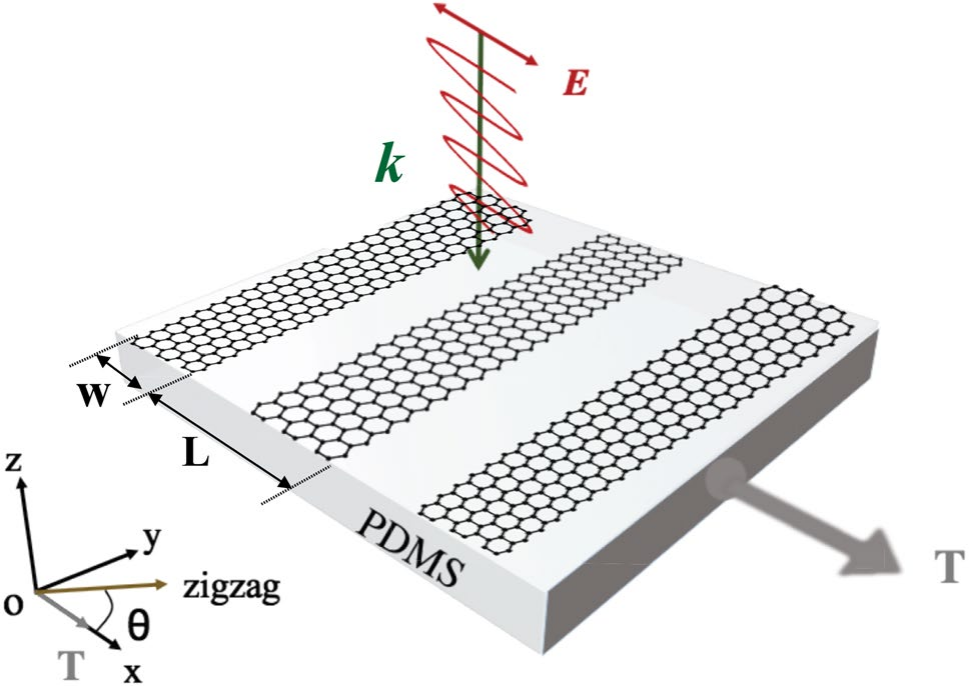
Schematic diagram of strain acting on graphene nanoribbons. A periodic array of graphene nanoribbons (PAGRs) of width W and period L was placed on flexible substrate PDMS, the angle between the zigzag direction of the honeycomb lattice of graphene and the *x*-axis is $\theta$, and the composited system is uniformly stretched along *Ox* axis direction.

Figure 1 depicts our studied system, the PAGRs with a period of *L* and width of *W* was placed on flexible substrate PDMS, while the composited system is uniformly stretched along a prescribed direction, the Cartesian system is chosen in the way that *Ox* coincides with the direction in which the strain is applied and the strain tensor reads^38^

1 $$\begin{aligned} {\overline{\kappa }}=\kappa \begin{pmatrix} \cos ^{2}\theta -\rho \sin ^{2}\theta & \quad (1+\rho )\cos \theta \sin \theta \\ (1+\rho )\cos \theta \sin \theta &\quad \sin ^{2}\theta -\rho \cos ^{2}\theta \end{pmatrix} \end{aligned}$$

where $\kappa$ is the strain modulus, $\theta$ denotes the angle between the tension *T* (*Ox* axis) and the zigzag direction and $\rho =0.165$ is known for Poisson’s ratio of graphite.

**Figure 2:**
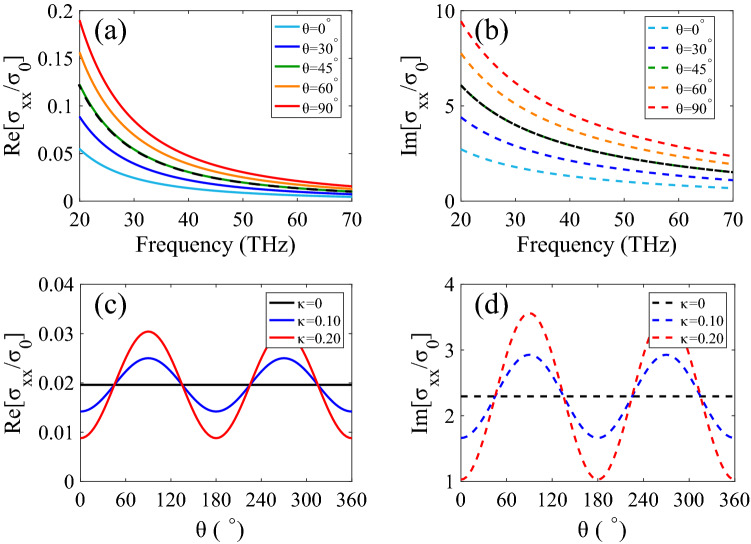
The anisotropic optical conductivity of graphene under strain engineering with the Fermi energy $E_F$ of 0.4 eV. (**a**),(**b**) The real and imaginary part of graphene conductivity along the x direction with a strain modulus of $\kappa =0.20$ for different angle $\theta$. (**c**),(**d**) The real and imaginary part of graphene conductivity along the x direction as a function of angle $\theta$ for the incident wave at 50 THz.

To demonstrate the mechanism of plasmon based graphene strain sensing, we first studied the conductivity of graphene under strain engineering. Figure 2a and b shows the real and imaginary part of graphene conductivity along the *x*-direction with a strain modulus of $\kappa =0.20$ for different angle $\theta$. At the same time, we have calculated the real and imaginary part of the unstrained case for comparison, as the dashed black line described in Fig. 2a and b. Then, how the real and imaginary part of graphene conductivity changes with the angle $\theta$ was depicted in Fig. 2c and d, where the incident wave has a frequency of 50 THz. Moreover, the conductivity of graphene with strain modulus $\kappa$ of 0.20 (red line) and 0.10 (blue line) are compared with the case of $\kappa =0$ (black line). One can see that the conductivity is anisotropic, at the same time, the real and imaginary part of graphene conductivity along the *x*-direction periodically alter with the change of angle $\theta$. In these above calculations, the Fermi energy $E_F$ is chosen as 0.4 eV.

To begin with, let’s focus on the period *L* of the PAGRs. The plasmon resonances in periodic arrays of graphene ribbons have been studied in detail by Nikitin et al.^43^. Their results show that the graphene plasmons in neighboring ribbons hybridize only for narrow gaps between the ribbons (gap width $$\le 0.2L$$), and the modes corresponding to different ribbons are very weakly coupled to each other. Besides, it has been reported that the transmission or absorption spectra for the arrays and the single ribbon are approximately equal. It should be noted that the coupling between the graphene nanoribbons does not need to be considered for strain sensing in this study. Therefore, the period of the PAGRs was set as $$ L=10 W$$ in the following calculations.

**Figure 3:**
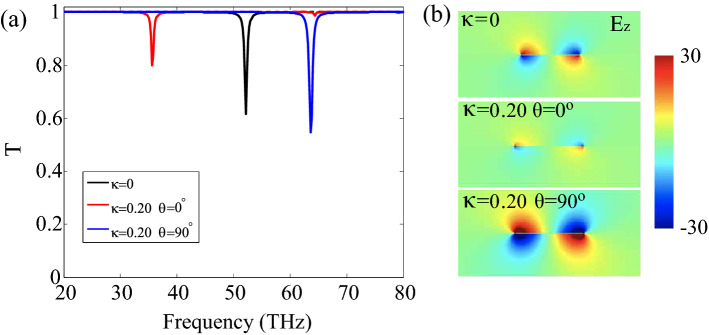
Surface plasmons excited by normally incident light in PAGRs, the width of ribbons is $W=50 \, \hbox {nm}$, the period of the PAGRs was set as $L=10 W$ and the Fermi energy $E_F$ is 0.4 eV. (**a**) The calculated transmission spectra *T* of self-standing PAGRs. The black, red and blue lines are corresponding to the unstrained PAGRs ($\kappa =0$), strained PAGRs ($\kappa =0.20$) with $\theta = 0^{\circ }$ and $\theta =90^{\circ }$ respectively. (**b**) The z-component of the electric near-field of plasmon modes.

Then, we turn to the plasmon resonances excited in the graphene ribbons with a width of 50 nm. For simplicity, the graphene is assumed to be self-standing with the Fermi energy $E_{F}= 0.4 \,\hbox {eV}$. The transmission spectrum *T* of unstrained and strained PAGRs are calculated in Fig. 3a, Here, the unstrained and strained graphene are represented by the strain modulus $\kappa =0$ and 0.20, respectively. For the unstrained PAGRs ($\kappa =0$, black line), one can see that the transmission spectrum *T* has a resonance frequency at 52.9 THz, which is consistent with previous work^41^. When the configuration is strained with a modulus $\kappa =0.20$, the position of the plasmon resonance redshifts (33.2 THz, the red line in Fig. 3a) for $\theta =0^{\circ }$. Otherwise, the resonance frequency blueshifts (59.6 THz, the blue line in Fig. 3a) for the case of $\theta =90^{\circ }$. It can be explained by the increase or decrease of strained graphene conductivity compared with unstrained graphene, which has shown in Fig. 2. Besides, the corresponding electric near-field of unstrained or strained PAGRs was calculated and demonstrated in Fig. 3b. A significant change in the resonance intensity can also be seen in the diagram. As a consequence, the shift of the localized graphene plasmon polariton resonance can be used to probe the strain stretching effectively.

**Figure 4:**
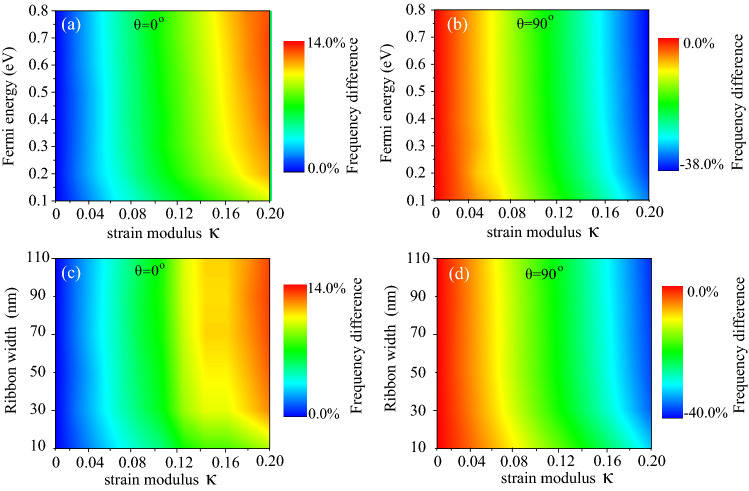
(**a**),(**b**) The differences in resonance frequencies $\Delta f$ dependence on Fermi energy and strain modulus for (**a**) $\theta =0^{\circ }$ and (**b**) $\theta =90^{\circ }$, the width of the ribbon is 50 nm. (**c**),(**d**) Ribbon width and strain modulus resolved the differences in resonance frequencies $\Delta f$ for the case of (**c**) $\theta =0^{\circ }$ and (**d**) $\theta =90^{\circ }$, where the Fermi energy $E_F=0.4 \, \hbox {eV}$.

Next, on account of the optical properties of graphene are parameter dependent, the Fermi energy $E_F$, ribbon width *W* dependent plasmon resonance of the unstrained and strained PAGRs is considered. Here the difference between strained and unstrained situations can be defined as the relative change in plasmon resonance frequency in terms of percentage $\Delta f=(f-f_0)/{f_0}\times 100\%$, where $f_0$ is the plasmon resonance frequency under zero strain; *f* is the resonance frequency under strain engineering. To begin with, the differences in resonance frequencies calculated for various Fermi energies and strain modulus is shown in Fig. 4a, where the width of the graphene ribbon is fixed at 50 nm; the angle between the zigzag direction of the graphene and the x-axis is set to $\theta =0^{\circ }$. We can see that the value of $\Delta f$ increases as the increasing of strain modulus and the maximum $\Delta f$ can reach about 14% for $\kappa =0.2$. It means that for the case of $\theta =0^{\circ }$, the plasmon resonance frequency will blueshift when strain engineering is applied and the shift can be up to 14% without damaging the structure of graphene. Moreover, with the increase of Fermi energy, the differences in resonance frequencies change little. Similar to Fig. 4a, b depicts the case of the angle $\theta =90^{\circ }$, one can find that the value of $\Delta f$ decreases as the increasing of strain modulus, and the maximum $\Delta f$ can reach to about 38% for $\kappa =0.2$, which means that the plasmon resonance frequency redshift for this case. Next, the width of the graphene ribbon and the strain modulus resolved $\Delta f$ is described in Fig. 4c,d. Here the Fermi energy of the graphene ribbon is fixed at 0.4 eV; Fig. 4c,d represented the case of $\theta =0^{\circ }$ and $\theta =90^{\circ }$, respectively. In accordance with Fig. 4a,b, with the strain modulus varies from 0 to 0.2, the value of $\Delta f$ increases up to 14% (in Fig. 4c) or decreases to 40% (in Fig. 4d), while the change in width of the ribbon hardly affects the differences in resonance frequencies for strained graphene ribbon compared to unstrained ribbon. Based on the above results, we can conclude that the Fermi energy $E_F$ or ribbon width *W* have no apparent influence on plasmon resonance frequencies difference when considering the strain engineering.

**Figure 5:**
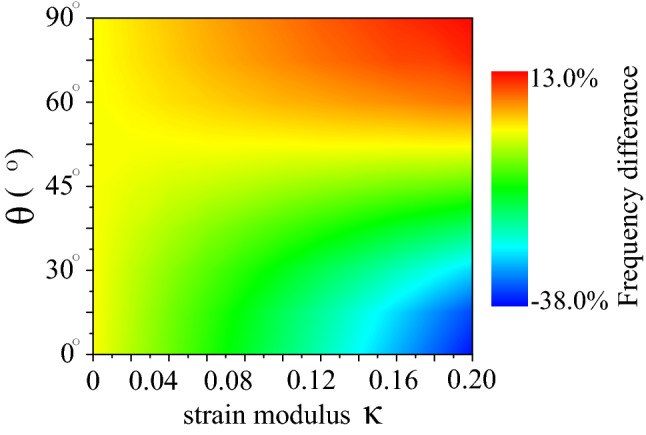
The differences in resonance frequencies of the nano ribbon resolved by various strain modulus $\kappa$ and different angle $\theta$, where the width of the ribbon is 50 nm and the Fermi energy of the ribbon is 0.4 eV.

In order to better illustrate the resonance frequencies controlled by strain engineering, the differences in resonance frequencies of the PAGRs resolved by various strain modulus $\kappa$ and different angle $\theta$ is plotted in Fig. 5. Here, the width and the Fermi energy of the ribbon are set to 50 nm and 0.4 eV respectively. One can see clearly that the value of $\Delta f$ changes from − 38 to 13 % as the strain modulus $\kappa$ and angle $\theta$ changes. Besides, with the increases of the strain modulus $\kappa$ of the PAGRs, the resonance frequency depends more on the angle between the zigzag direction of the graphene and the *x*-axis. This can be traced back to the anisotropic conductivity of graphene under strain engineering as shown in Fig. 2. It’s worth mentioning when the angle between the zigzag direction of the graphene and the *x*-axis is $45^{\circ }$, the plasmon resonance peak of the graphene ribbon does not shift. It can be explained by the conductivity of graphene under strain engineering for $\theta =45^{\circ }$ equals to the conductivity of unstrained graphene as shown in Fig. 2. Therefore, we can draw a conclusion that the shift of the plasmon resanonc in PAGRs is only sensitive to the modulus $\kappa$ and direction $\theta$ of strain applying, which will beneficial for strain sensing.

**Figure 6:**
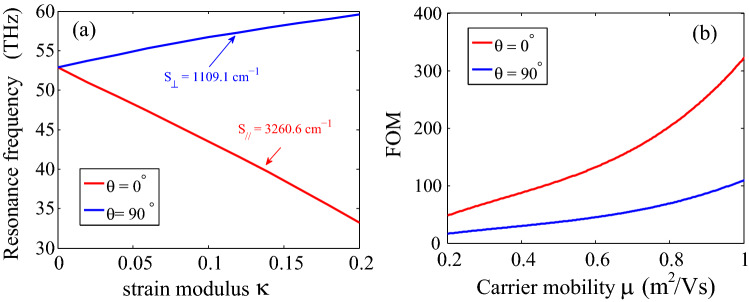
(**a**) The sensitivity, and (**b**) The FOM-factor of plasmon resonance based strain sensors for the case of $\theta =0^{\circ }$ and $\theta =90^{\circ }$ respectively.

The sensitivity and figure of merit (FOM) were calculated to qualitatively evaluate the performance of the proposed strain sensor. Sensitivity is the electronic optical response (or output) signal variation $U_{out}$ to an external physical input signal $U_{in}$ from a sensor, which defined as $S=\Delta U_{out}/\Delta U_{in}$. Considering our sensor, the associated sensitivity can be calculated by the relation:

$$\begin{aligned} S={(f-f_0)}/{\Delta \kappa } \end{aligned}$$

where $f-f_0$ represents the displacements of resonance frequency, and $\Delta \kappa$ represents the relative deformation of graphene under strain stretching. Figure 6a shows the change in frequency of graphene plasmon resonance with various strain modulus. Here the width of the ribbon is set as $W=50 \, \hbox {nm}$, and the Fermi energy $E_F =0.4 \, \hbox {eV}$. Then, the sensitivity of the proposed sensor can be obtained by calculating the slope of the line in Fig. 6a. When tension direction is parallel to the zigzag direction ($\theta =0^{\circ }$), the sensitivity was calculated to be $S_{//} =3260.6 \, {\hbox {cm}}^{-1}$, while it was $S_{\perp } = 1109.1 \, {\hbox {cm}}^{-1}$ for the perpendicular $\theta =90^{\circ }$. FOM is another important parameter to assess the performance of the sensor, which can be defined as FOM = S/FWHM. here FWHM is the Full Width of Half Maximum of surface plasmon resonance depends on the carrier mobility of graphene. The dependence of the FOM factor on mobility was described in Fig. 6b. One can see that when the carrier mobility of graphene changes from 2,000 to 10,000 cm$^2$/Vs, the value of FOM ranging between 50 and 320 for the case of $\theta =0^{\circ }$, while the value varies from 10 to 110 for $\theta =90^{\circ }$. Higher FOM-factor leads to higher detection accuracy. In the proposed graphene plasmon based strain sensor, no matter which direction the graphene is stretched, the FOM-factors are all above 10 even if the graphene has a low carrier. That is, our proposed sensor could be potentially useful from a practical point of view.

**Figure 7:**
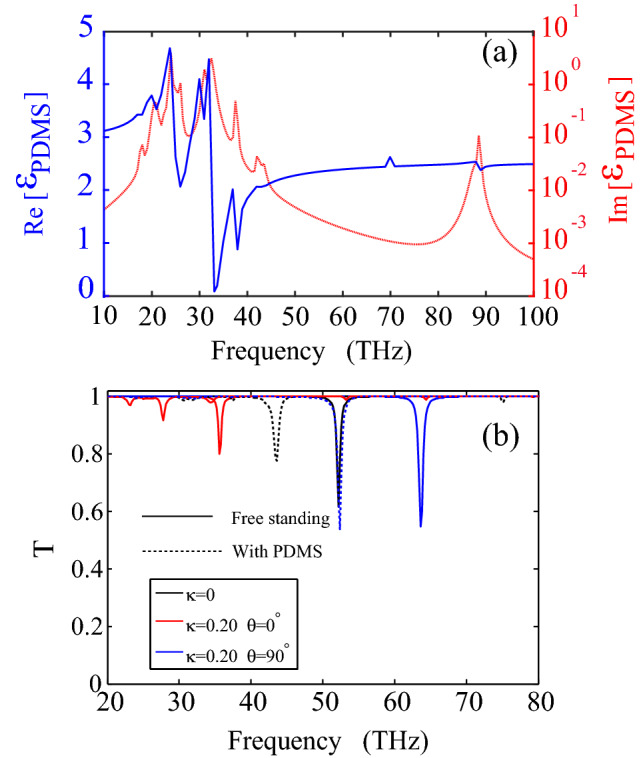
(**a**) Real (solid blue line, left y-axis) and imaginary (dotted red line, right y-axis) parts of the complex permittivity of PDMS. (**b**) The PDMS dependent plasmon resonances in PAGRs.

At last, all the above calculations are based on self-standing PAGRs, and it is well known that the plasmon resonance depends a lot on the dielectric environment due to its evanescent property. The configuration should be placed on a flexible substrate (PDMS or PET) in real strain-based experiments. Take PDMS as an example, the optical properties of PDMS between 10 and 100 THz was taken from a Drude–Lorentz oscillator model with 15 oscillators, which is given by

2 $$\begin{aligned} \epsilon (\omega )=\epsilon _{\infty }+\sum _{k=1}^{N} \left[ \frac{s_k}{1-\left( \frac{\omega }{\omega _k}\right) ^2 -i\Gamma _k \left( \frac{\omega }{\omega _k}\right) }\right] \end{aligned}$$

where $\epsilon _{\infty }=2.276$, the value of $\omega _k$, $s_k$ and $\Gamma _k$ was taken from Ref.^42^. Figure 7a shows the real (solid blue line) and imaginary (dotted red line) parts of the complex permittivity of PDMS, respectively. In Fig. 7b, the plasmon responses of PAGRs with (dashed line) and without PDMS (solid line) for three different cases are compared, the three cases are unstrained graphene ribbon $\kappa =0$ (black line), strained ribbon $\kappa =0.20$ with the angle of $\theta =0^{\circ }$ (red line) and $\theta =90^{\circ }$ (blue line). The width and the Fermi energy of the ribbon set to 50 nm and 0.4 eV. One can see the plasmon resonance frequency redshifts both for the three cases. Moreover, the frequency difference between strained and unstrained graphene ribbon is almost the same whether the graphene ribbon was placed on PDMS or not, which means that the strain sensing is also kept when the substrate is considered.

We can get significant information from the above analysis about which parameter, including the carrier mobility Fermi energy $E_F$ of graphene, ribbon width *W* of the proposed PAGRs, strain modulus $\kappa$ and strain direction $\theta$, should be paid more attention in specific experiments. The theoretical research of the proposed surface plasmon resonance based graphene strain sensor, not only providing a new optical method for graphene strain sensing, but also laying a foundation for the far-field experimental detection. At the same time, it also lays a theoretical foundation for further near-field research based on graphene plasmons under strain stretching.

## Discussion

In summary, we have explored the optical response of PAGRs under elastic deformations in mid-infrared range, and the highly localized plasmon resonances in PAGRs are sensitive to external strain stretching. The external strain can be effectively detected by the frequency shift of the plasmon resonances. Our results show that the Fermi energy $E_F$, and ribbon width *W* has no apparent influence on the frequency shift of plasmon resonance, while it depends more on the angle $\theta$ and the strain modulus $\kappa$. On the other hand, high FOM can be obtained for the proposed sensor by taking advantage of the high localization of graphene plasmons in graphene nanoribbons. The calculated FOM in the proposed configuration can up to two orders of magnitude, which could be useful in future applications. In a word, the strain stretching can be effectively and sensitively detected by the frequency shift of the localized graphene plasmon resonances, and this realization opens up a simple and viable avenue for strain sensing.

## Methods

### Conductivity of graphene under strain engineering

The frequency dependent conductivity tensor of graphene under uniform strain can be written as^35^

3 $$\begin{aligned} {\widetilde{\sigma }}(\omega )=\sigma (\omega )({\overline{I}}-2\beta {\overline{\kappa }} +\beta {{\text {Tr}}({\overline{\kappa }})}) \end{aligned}$$

where $\beta \simeq 2.37$ is related to Grüneisen parameter^36,39^. Moreover, in the absence of electron-electron interaction, the band gap of graphene cannot be opened in a pure tight-binding model for deformation $\kappa$ under 20% ($\kappa \simeq 0.20$)^5^. In addition, the isotropic optical conductivity of graphene $\sigma (\omega )$ can be computed within the local-random phase approximation (RPA)^40^, which is written as

4 $$\begin{aligned} \sigma (\omega )= & \, \frac{2e^2T}{\pi \hbar }\frac{i}{\omega +i\tau ^{-1}} \log \Bigg [2\cosh \Bigg (\frac{E_F}{2K_BT}\Bigg )\Bigg ]\nonumber \\&+\frac{e^2}{4\hbar }\Bigg [H(\omega /2)+\frac{4i\omega }{\pi } \int _{0}^{\infty }d\varepsilon \frac{H(\varepsilon )-H(\omega /2)}{\omega ^2-4\varepsilon ^2}\Bigg ], \nonumber \\ \end{aligned}$$

where

$$\begin{aligned} H(\varepsilon )=\frac{\sinh (\hbar \varepsilon /k_BT)}{\cosh (E_F/k_BT) +\cosh (\hbar \varepsilon /k_BT)}. \end{aligned}$$

The intrinsic relaxation time $\tau$ is estimated from $\tau =\mu E_F/ev_F^2$, where $v_F\approx c/300$ is the Fermi velocity and $\mu =10,000 \,{\hbox {cm}}^2/\hbox {Vs}$ is the measured DC mobility. Here the ambient temperature *T* is set as 300 K.

### Details of simulation

The optical response of the designed structures is obtained by using the finite element method. Because of translation invariance along y-axis, only the two-dimensional model was considered in our simulation. To achieve plasmon resonance, the incident plane wave is chosen to be *x*-polarized and propagates along *z*-axis with unit electric amplitude $E = 1 \, \hbox {V/m}$. Although the conductivity of graphene will exhibit in-plane anisotropy under strian engineering, only the conductivity along the polarization direction (*x*-axis) need to be considered. In the simulations, the effective dielectric constant of graphene is $\epsilon _{g}(\omega )= 1+i{\sigma _{xx}} (\omega )/(\omega \epsilon _0d)$, where $d=0.5 \, \hbox {nm}$ as a reasonable value close to the $d\rightarrow 0$ limit.

## References

[1] Novoselov KS, Geim AK, Morozov SV, Jiang D, Zhang Y, Dubonos SV, Grigorieva IV, Firsov AA (2004). Electric field effect in atomically thin carbon films. Science.

[2] Geim AK, Novoselov KS (2007). The rise of graphene. Nat. Mater..

[3] Geim AK (2009). Graphene: status and prospects. Science.

[4] Bolotin KI, Sikes KJ, Jiang Z, Klima M, Fudenberg G, Hone J, Kim P, Stormer HL (2008). Ultrahigh electron mobility in suspended graphene. Solid State Commun..

[5] Lee C, Wei X, Kysar JW, Hone J (2008). Measurement of the elastic properties and intrinsic strength of monolayer graphene. Science.

[6] Heersche HB, Pablo JH, Oostinga JB, Vandersypen LMK, Morpurgo AF (2007). Bipolar supercurrent in graphene. Nature.

[7] Becerril HA, Jie M, Zunfeng L, Stoltenberg RM, Zhenan B, Yongsheng C (2008). Evaluation of solution-processed reduced graphene oxide films as transparent conductors. ACS Nano.

[8] Zhu Y, Murali S, Stoller MD, Velamakanni A, Piner RD, Ruoff RS (2010). Microwave assisted exfoliation and reduction of graphite oxide for ultracapacitors. Carbon.

[9] Yi W, Rong Y, Zhiwen S, Lianchang Z, Dongxia S, Enge W, Guangyu Z (2011). Super-elastic graphene ripples for flexible strain sensors. ACS Nano.

[10] Rodrigo D, Limaj O, Janner D, Etezadi D, Abajo FJGD, Pruneri V, Altug H (2015). Mid-infrared plasmonic biosensing with graphene. Science.

[11] Varghese SS, Varghese SH, Swaminathan S, Singh KK, Mittal V (2015). Two-dimensional materials for sensing: graphene and beyond. Electronics.

[12] Marini A, Silveiro I, Abajo FJGD (2018). Molecular sensing with tunable graphene plasmons. ACS Photonics.

[13] Wu S, He Q, Tan C, Wang Y, Zhang H (2013). Graphene-based electrochemical sensors. Small.

[14] Cao K, Feng S, Han Y, Gao L, Ly TH, Xu Z, Lu Y (2020). Elastic straining of free-standing monolayer graphene. Nat. Commun..

[15] Chun S, Choi Y, Park W (2017). All-graphene strain sensor on soft substrate. Carbon.

[16] Zhao J, He C, Yang R, Shi Z, Cheng M, Yang W, Xie G, Wang D, Shi D, Zhang G (2017). Ultra-sensitive strain sensors based on piezoresistive nanographene films. Appl. Phys. Lett..

[17] Bae S-H, Lee Y, Sharma BK, Lee H-J, Kim J-H, Ahn J-H (2013). Graphene-based transparent strain sensor. Carbon.

[18] Lee Y, Bae S, Jang H, Jang S, Zhu S-E, Sim SH, Song YI, Hong BH, Ahn J-H (2010). Wafer-scale synthesis and transfer of graphene films. Nano Lett..

[19] Tian H, Shu Y, Cui Y-L, Mi W-T, Yang Y, Xie D, Ren T-L (2014). Scalable fabrication of high-performance and flexible graphene strain sensors. Nanoscale.

[20] Wan S, Bi H, Zhou Y, Xie X, Su S, Yin K, Sun L (2017). Graphene oxide as high-performance dielectric materials for capacitive pressure sensors. Carbon.

[21] Berger C, Phillips R, Centeno A, Zurutuza A, Vijayaraghavan A (2017). Capacitive pressure sensing with suspended graphene-polymer heterostructure membranes. Nanoscale.

[22] Luo S, Yang J, Song X, Zhou X, Yu L, Sun T, Yu C, Huang D, Du C, Wei D (2018). Tunable-sensitivity flexible pressure sensor based on graphene transparent electrode. Solid State Electron..

[23] Jun M, Wei J, Hoi Lut H, Ji Yan D (2012). High-sensitivity fiber-tip pressure sensor with graphene diaphragm. Opt. Lett..

[24] Ma J, Ju J, Jin L, Jin W (2011). A compact fiber-tip micro-cavity sensor for high-pressure measurement. IEEE Photonic Technol. Lett..

[25] Lee S, Reuveny A, Reeder J, Lee S, Jin H, Liu Q, Yokota T, Sekitani T, Isoyama T, Abe Y (2016). A transparent bending-insensitive pressure sensor. Nat. Nanotechnol..

[26] Tiefenauer RF, Dalgaty T, Keplinger T, Tian T, Shih C-J, Vörös J, Aramesh M (2018). Monolayer graphene coupled to a flexible plasmonic nanograting for ultrasensitive strain monitoring. Small.

[27] Xu Y, Guo Z, Chen H, Yuan Y, Lou J, Lin X, Gao H, Chen H, Yu B (2011). In-plane and tunneling pressure sensors based on graphene/hexagonal boron nitride heterostructures. Appl. Phys. Lett..

[28] Trung TQ, Tien NT, Kim D, Jang M, Yoon OJ, Lee N-E (2014). A flexible reduced graphene oxide field-effect transistor for ultrasensitive strain sensing. Adv. Funct. Mater..

[29] Shin S-H, Ji S, Choi S, Pyo K-H, An BW, Park J, Kim J, Kim J-Y, Lee K-S, Kwon S-Y (2017). Integrated arrays of air-dielectric graphene transistors as transparent active-matrix pressure sensors for wide pressure ranges. Nat. Commun..

[30] Sun S, Guo L, Chang X, Liu Y, Niu S, Lei Y, Liu T, Hu X (2019). A wearable strain sensor based on the zno/graphene nanoplatelets nanocomposite with large linear working range. J, Mater. Sci..

[31] Setare M, Majari P, Noh C, Dehdashti S (2019). Photonic realization of the deformed dirac equation via the segmented graphene nanoribbons under inhomogeneous strain. J. Mod. Opt..

[32] Ni ZH, Yu T, Lu YH, Wang YY, Feng YP, Shen ZX (2008). Uniaxial strain on graphene: Raman spectroscopy study and band-gap opening. ACS Nano.

[33] Vitor MP, Ribeiro RM, Peres NMR, Neto AHC (2010). Optical properties of strained graphene. Europhys. Lett..

[34] Pellegrino FMD, Angilella GGN, Pucci R (2010). Strain effect on the optical conductivity of graphene. Phys. Rev. B.

[35] Oliva-Leyva M, Gerardo GN (2014). Anisotropic ac conductivity of strained graphene. J. Phys. Condens. Matter..

[36] Oliva-Leyva M, Naumis GG (2013). Understanding electron behavior in strained graphene as a reciprocal space distortion. Phys. Rev. B.

[37] Ma Z, Cai W, Xiang Y, Ren M, Zhang X, Xu J (2017). Dynamic spontaneous emission control of an optical emitter coupled to plasmons in strained graphene. Opt. Exp..

[38] Pereira VM, Castro Neto AH, Peres NMR (2009). Tight-binding approach to uniaxial strain in graphene. Phys. Rev. B.

[39] Blakslee OL, Proctor DG, Seldin EJ, Spence GB, Weng T (1970). Elastic constants of compression annealed pyrolytic graphite. J. Appl. Phys..

[40] Wunsch B, Stauber T, Sols F, Guinea F (2006). Dynamical polarization of graphene at finite doping. New J. Phys..

[41] Christensen J, Manjavacas A, Thongrattanasiri S, Koppens FHL, García de Abajo FJ (2012). Graphene plasmon waveguiding and hybridization in individual and paired nanoribbons. ACS Nano.

[42] Srinivasan A, Czapla B, Mayo J, Narayanaswamy A (2016). Infrared dielectric function of polydimethylsiloxane and selective emission behavior. Appl. Phys. Lett..

[43] Nikitin GFG-VFJ, Yu A, Martin-Moreno L (2011). Surface plasmon enhanced absorption and suppressed transmission in periodic arrays of graphene ribbons. Phys. Rev. B.

